# EAACI anaphylaxis guidelines: systematic review protocol

**DOI:** 10.1186/s13601-020-00320-3

**Published:** 2020-05-20

**Authors:** Debra de Silva, Graham Roberts, Margitta Worm, Antonella Muraro, the EAACI Food Allergy and Anaphylaxis Guidelines Group, Cherry Alviani, Elizabeth Angier, Kirsten Beyer, M. Beatrice Bilo, Carsten Bindslev-Jensen, Knut Brockow, Victoria Cardona, Debra de Silva, Audrey DunnGalvin, Montserrat Fernandez-Rivas, Lene Heise Garvey, Susanne Halken, Britt Jensen, Ekaterina Khaleva, Louise J. Michaelis, Antonella Muraro, Hanneke Oude Elberink, Lynne Regent, Carmen Riggioni, Graham Roberts, Angel Sanchez, Chris Singh, Berber Vlieg-Boerstra, Margitta Worm

**Affiliations:** 1 The Evidence Centre Ltd, London, UK; 2 NIHR Southampton Biomedical Research Centre, University Hospital Southampton NHS Foundation Trust, Southampton, UK; 3 Clinical and Experimental Sciences and Human Development in Health, Faculty of Medicine, University of Southampton, Southampton, UK; 4 The David Hide Asthma and Allergy Research Centre, St Mary’s Hospital, Isle of Wight, UK; 5 Division of Allergy and Immunology, Dpt Dermatology, Venerology and Allergy, Charité Universitätsmedizin Berlin, Berlin, Germany; 6 Food Allergy Referral Centre Veneto Region, Department of Women and Child Health, Padua General University Hospital, Padua, Italy

**Keywords:** Anaphylaxis, Prevention, Management, Diagnosis, Adrenaline, Epinephrine

To the editor,

Anaphylaxis is a clinical emergency that all healthcare professionals and teachers should be able to recognise, manage and help prevent. In Europe, about one in 300 people will experience anaphylaxis at some time in their lives [1]. Rapid and effective care helps to keep the overall fatality rate low, but much is uncertain about the most effective ways to prevent, diagnose and manage anaphylaxis.

In 2014 the European Academy of Allergy and Clinical Immunology (EAACI) published guidelines about the acute and long-term management of anaphylaxis [2]. This is a rapidly developing field so the guidelines are being updated to reflect the latest evidence. In 2020 a systematic review will be undertaken to inform the guidelines. The review will examine the effectiveness of approaches for the diagnosis, acute management and prevention of anaphylaxis in children and adults.

This letter briefly sets out key elements of the systematic review protocol. Full details are available on the International Prospective Register of Systematic Reviews (PROSPERO number CRD42019159739).

A number of other systematic reviews have examined anaphylaxis. However the EAACI guidelines cannot be based on these alone because past reviews do not consider all interventions and outcomes of interest and they sometimes focus exclusively on care in acute hospitals. Some reviews are more than a decade old.

To alleviate gaps in available reviews, EAACI has drawn together an expert task force comprising allergists, anaesthetists, emergency medicine clinicians, paediatricians, pharmacists, primary care doctors, psychologists, paramedics, other clinicians, patient representatives and methodologists from seven countries. The group used a brainstorming and consensus process to agree the review questions and outcomes of interest:

- Population: children (aged under 18 years) and/or adults (18+ years) with or without a history of anaphylaxis.
- Intervention: any intervention to prevent, diagnose in an emergency or manage anaphylaxis in the community or hospital.
- Comparator: any comparator, including placebo, no intervention or any intervention or combination of interventions.
- Outcomes: anaphylaxis incidence, sensitivity and specificity of diagnostic approaches, mortality or near fatal incidents, biphasic reactions, quality of life, knowledge of anaphylaxis management, effectiveness of adrenaline administration.
- Study types: randomised controlled trials, controlled clinical trials, controlled before-and-after studies and case–control studies in humans and, in the case of diagnosis and adrenaline (epinephrine) only, consecutive case series with a minimum of 20 participants. There will be no language or geographical restrictions.

An information specialist will search five bibliographic databases for studies published between 1946 and 20 April 2020. Studies about immunotherapy will not be eligible as this is covered in other EAACI guidelines.

The Grading of Recommendations, Assessment, Development and Evaluation (GRADE) approach will be used to assess the certainty of the evidence [3]. The risk of bias in individual studies will be assessed using Cochrane-approved tools (ROB-2, ROBINS-I, QADAS-2). All data extraction and quality appraisal will be undertaken independently by two reviewers in partnership with a task force of clinicians and patient representatives.

Due to the heterogeneous nature of interventions, settings and populations, the reviewers expect to undertake a narrative synthesis, summarising the results about each intervention descriptively and drawing attention to any differences in outcome based on setting or demographics. If both randomised and non-randomised studies are available about any intervention, the narrative synthesis will divide the results for experimental versus observational data.

If the following criteria are met, random-effects meta-analysis will be undertaken: six or more studies about an outcome are available; studies provide sufficiently detailed quantitative data to allow compilation; the data available are measured in a similar manner; the populations have a similar demographic and condition profile, and no other relevant meta-analysis has been published. These criteria have been selected to ensure that any quantitative synthesis is not collating information from dissimilar studies and that the number of studies is sufficient to warrant collation. The minimum number of six studies has been selected because small studies and non-randomised designs are eligible for inclusion in the review. Random-effects modelling has been selected to avoid overweighting studies with the largest samples.

The EAACI task force will use the systematic review to help shape new guidelines, due for release in 2021. Disseminating the information from guidelines has been found to increase healthcare professionals’ knowledge about anaphylaxis [4]. But implementing guidelines can be challenging [5]. One of the key issues supporting the implementation of guidelines may be their scientific credibility. Ensuring that this systematic review uses a rigorous and consistent methodology will support up-to-date recommendations to guide national policy and individual practice.

Even so, randomised controlled trials are not usual for rare life-threatening conditions such as anaphylaxis and as a consequence the certainty of evidence in this field is likely to be low. EAACI’s guidelines process, in line with the GRADE approach, will supplement research about effectiveness with expert opinion and weigh up benefits against harms, costs, preferences and acceptability in developing final recommendations.

## Data Availability

Data sharing is not applicable to this article as no datasets were generated or analysed during the current study. Full details of the systematic review protocol are available on the International Prospective Register of Systematic Reviews (PROSPERO number CRD42019159739).

## Abbreviations

EAACI: European Academy of Allergy and Clinical Immunology
